# Early neonatal mortality and neurological outcomes of neonatal resuscitation in a resource-limited setting on the Thailand-Myanmar border: A descriptive study

**DOI:** 10.1371/journal.pone.0190419

**Published:** 2018-01-05

**Authors:** Sophie Janet, Verena I. Carrara, Julie A. Simpson, Nant War War Thin, Wah Wah Say, Naw Ta Mlar Paw, Kesinee Chotivanich, Claudia Turner, Jane Crawley, Rose McGready

**Affiliations:** 1 Centre for Tropical Medicine and Global Health, Nuffield Department of Medicine, University of Oxford, Old Road Campus, Oxford, United Kingdom; 2 Shoklo Malaria Research Unit, Mahidol-Oxford Tropical Medicine Research Unit, Faculty of Tropical Medicine, Mahidol University, Mae Sot, Thailand; 3 Centre for Epidemiology and Biostatistics, Melbourne School of Population and Global Health, The University of Melbourne, Melbourne, VIC, Australia; 4 Mahidol-Oxford Tropical Medicine Research Unit, Faculty of Tropical Medicine, Mahidol University, Bangkok, Thailand; 5 Cambodia-Oxford Medical Research Unit, Angkor Hospital for Children, Siem Reap, Cambodia; 6 Angkor Hospital for Children, Siem Reap, Cambodia; TNO, NETHERLANDS

## Abstract

**Background:**

Of the 4 million neonatal deaths worldwide yearly, 98% occur in low and middle-income countries. Effective resuscitation reduces mortality and morbidity but long-term outcomes in resource-limited settings are poorly described. This study reports on newborn neurological outcomes following resuscitation at birth in a resource-limited setting where intensive newborn care including intubation is unavailable.

**Methods:**

Retrospective analysis of births records from 2008 to 2015 at Shoklo Malaria Research Unit (SMRU) on the Thailand-Myanmar border.

**Findings:**

From 21,225 newbonrs delivered, 15,073 (71%) met the inclusion criteria (liveborn, singleton, ≥28 weeks’ gestation, delivered in SMRU). Neonatal resuscitation was performed in 460 (3%; 422 basic, 38 advanced) cases. Overall early neonatal mortality was 6.6 deaths per 1000 live births (95% CI 5.40–8.06). Newborns receiving basic and advanced resuscitation presented an adjusted rate for death of 1.30 (95%CI 0.66–2.55; p = 0.442), and 6.32 (95%CI 3.01–13.26; p<0.001) respectively, compared to newborns given routine care. Main factors related to increased need for resuscitation were breech delivery, meconium, and fetal distress (p<0.001). Neurodevelopmental follow-up to one year was performed in 1,608 (10.5%) of the 15,073 newborns; median neurodevelopmental scores of non-resuscitated newborns and those receiving basic resuscitation were similar (64 (n = 1565) versus 63 (n = 41); p = 0.732), while advanced resuscitation scores were significantly lower (56 (n = 5); p = 0.017).

**Interpretations:**

Newborns requiring basic resuscitation at birth have normal neuro-developmental outcomes at one year of age compared to low-risk newborns. Identification of risk factors (e.g., breech delivery) associated with increased need for neonatal resuscitation may facilitate allocation of staff to high-risk deliveries. This work endorses the use of basic resuscitation in low-resource settings, and supports on-going staff training to maintain bag-and-mask ventilation skills.

## Introduction

Birth is one of the most challenging biological events in the human life cycle. According to a 2005 World Health Organization report, around 136 million newborns are born each year[1] of which 10 million (5–10%) need assistance to initiate effective breathing.[2] Basic resuscitation with bag-and-mask ventilation is needed in approximately 6 million newborns each year. Advance resuscitation is needed in less than 1%. [3]

Globally 4 million neonatal deaths are registered annually, representing 40% of under-5 child mortality.[1] Seventy-three percent of neonatal deaths occur in the first week of life, especially during the first 24 hours after delivery.[4] Ninety-eight percent of all neonatal deaths occur in low- and middle-income countries, and 77% of those in Asia and Sub-Saharan Africa.[5]

Over a million children who survive birth hypoxia each year develop problems such as cerebral palsy, learning difficulties and other disabilities.[1] The economic impact of the morbidities related to birth hypoxia in 2004 accounted for 41,683,855 disability-adjusted life years worldwide.[1] Many low-income countries lack any support services for these children.

These data emphasise the need to provide early quality healthcare to newborns born in poor condition.

It is estimated that between 6% to 42% of neonatal mortality or morbidity in low-income countries could be avoided by basic neonatal resuscitation.[6] Furthermore, a meta-analysis showed that provision of neonatal resuscitation training in facilities reduced intrapartum-related deaths in term newborns by 30%.[2] Increasing neonatal resuscitation coverage to 90% of the deliveries currently taking place in health facilities would save more than 93,000 newborn lives each year.[7]

Gestational age at birth is a further determinant of neonatal outome,[8] yet no clear threshold for resuscitation has been defined in the literature. The reality in low-resource settings is that extremely preterm newborns (<28 weeks gestational age) are not generally resuscitated. Survival improves with every added week of gestational age, and moderate to late preterm (>32 to <37 weeks) newborns have higher probabilities of survival with basic neonatal resuscitation.[9] Identifying the newborns most likely to require resuscitation at birth is critical to effective planning and provision of appropriate care,[10] although some newborns will have no identifiable risk factors.[10]

Neonatal resuscitation using basic equipment and skills has been shown to be feasible and effective in resource-limited settings, but the long-term outcome of newborns undergoing basic or advanced neonatal resuscitation in such settings is unclear.[6]^,^[11]

The primary objectives of the study were to describe early neonatal mortality and neurodevelopmental outcome at one year of age of newborns who received basic, advanced or no neonatal resuscitation after birth at SMRU. Secondary objectives were to identify perinatal risk factors associated with newborn resuscitation, and to identify baseline characteristics predicting poor neonatal outcomes.

## Methods

### Study design and participants

This was a retrospective analysis of hospital records with data spanning the antenatal period, birth, and admission to the special care baby unit of the Shoklo Malaria Research Unit (SMRU) located at the Thailand-Myanmar border (supportive information, Text A in S1 File). Liveborn, singleton newborns of ≥28 weeks’ gestation (determined by ultrasound scan or Dubowitz examination in case of late scan [12]) who were born at SMRU between 1 January 2008 and 31 December 2015 were included in the study. Stillborns, newborns from multiple pregnancies (<0.9%), and newborns with major congenital abnormalities were excluded.

**Fig 1:**
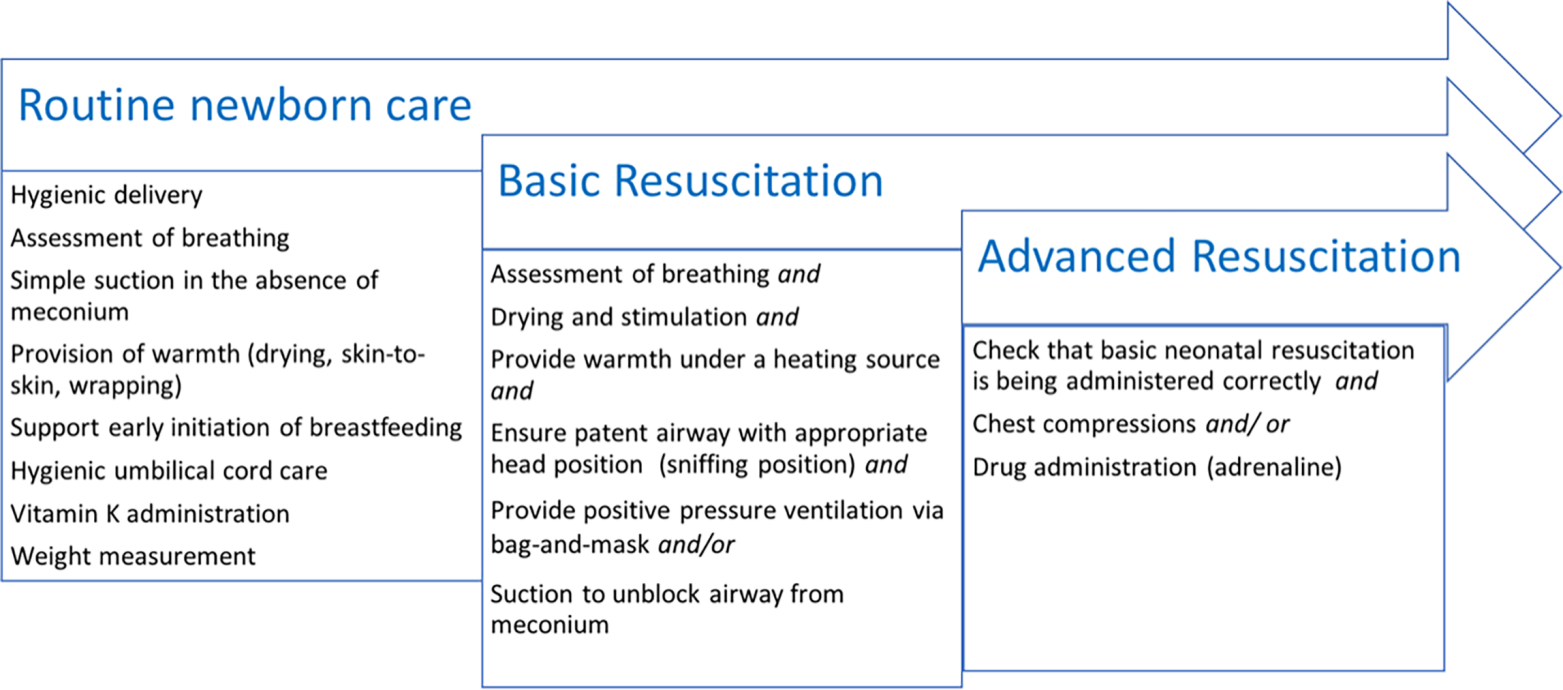
Definitions of different levels of newborn assistance at birth in SMRU.

Newborn resuscitation training and implementation was standardized by a UK trained paediatrician (author: CT) and introduction of Advanced Life Support in Obstetrics (ALSO®) course [13] at SMRU in 2008. The ALSO® is one of several evidence-based inter-professional learning programs that assist health professionals develop and maintain the knowledge and skills required to manage emergencies that may arise during childbirth. Simple oral suction carried out for any other reason other than unblocking the airway from thick meconium was classified as routine newborn care. Basic resuscitation was defined as ventilation via bag-and-mask and/or suction to unblock the airway from thick meconium or other secretions. Ventilation via endotracheal tube was unavailable. Transfer to a higher level care for intubation and ventilation is a major financial constraint for health non-governmental organizations in this region and is not routinely available for marginalized populations (refugees and migrant) with whom these organizations work. Advanced resuscitation was defined as the need for chest compressions and/or administration of intravenous (or subcutaneous) adrenaline following unsuccessful basic resuscitation (Fig 1) (supportive information, Text B in S1 File).

### Variables included in the study

Baseline data (Table 1) on all mothers and newborns were collected from the antenatal electronic records, and were selected on the basis of their availability in the records and their medical importance, supported by the published literature (supportive information, Text C in S1 File).

**Table 1:** Maternal and Newborn characteristics by groups of neonatal resuscitation from 2008 to 2015.

Maternal characteristics	No Resuscitation N = 14,613	Basic N = 422	Advanced N = 38
Age y, mean (SD, min-max)	25.9 (6.6, 14–53)	24.7 (6.8, 15–43)	24.7 (7.3, 15–47)
*BMI kg/m^2^, mean (SD, min-max)	22.1 (3.3, 13.2–42.1)	22.2 (3.3, 15.8–39.2)	23.1 (4.3, 17.2–37.4)
BMI <18.5, n (%)	1,463/14,271 (10.3)	45/412 (11.2)	3/35 (8.6)
BMI 18.5–< 23.0, n (%)	8,202/14,271 (57.5)	228/412 (55.3)	19/35 (54.3)
BMI ≥23.0, n (%)	4624/14,271 (32.4)	139/412 (33.7)	13/35 (37.1)
Primigravida, n (%)	4,859 (33.2)	219 (51.9)	23 (60.5)
Migrant, n (%)	6,816/14,612 (46.6)	183 (43.3)	14 (36.8)
Literate, n (%)	7,413/11,821 (62.7)	206/322 (63.9)	16/25 (64.0)
Smoking, n (%)	2,085 (14.3)	45 (10.7)	4 (10.5)
Anaemia, n (%)	609/14,579 (4.2)	15 (3.5)	1 (2.6)
Any maternal hypertension n, (%)	638 (4.4)	32 (7.6)	4 (10.5)
Malaria n, (%)	1,121/ 14,610 (7.7)	30/416 (7.2)	2/37 (5.4)
Gestational diabetes, n (%)	135/ 7,520 (1.8)	4/185 (2.1)	1/17 (5.8)
Previous neonatal death, n (%)	4,234/ 14,569 (29.0)	112/413 (27.1)	10/37 (27.0)
**Delivery Characteristics**			
*APH, n (%)	36/14,596 (0.2)	2/415 (0.48)	0/37
Breech delivery, n (%)	155 (1.1)	64 (15.2)	9 (23.7)
Prolonged 2^nd^ stage, n (%)	886 (6.0)	79 (15.2)	7 (18.4)
*PROM, n (%)	604/ 14,218 (4.2)	31/405 (9.1)	7/36 (19.4)
Maternal fever, n (%)	214/14,584 (1.5)	18/416 (4.3)	5/37 (13.5)
Foetal distress, n (%)	580 (3.9)	111 (26.3)	11 (28.9)
Meconium, n (%)	3,497 (23.9)	205 (48.6)	21 (55.3)
*EGA wks, mean (SD, min-max)	39.0 (1.5, 28–43.5)	38.6 (2.6, 28–42.3)	36.9 (3.6, 28.6–41.1)
*PTB, n (%)	669 (4.6)	42 (10.0)	9 (31.0)
Male n, (%)	7,514/ 14,612 (51.4)	258 (61.1)	20 (52.6)
*BW g, mean (SD, min-max)	2969 (444, 565–5230)	2867 (591, 960–4635)	2357 (773, 740–4264)
*BW <2500g, n (%)	1,772 (12.1)	84 (20.0)	20 (52.6)
*LGA, n (%)	268 (1.8)	12 (2.8)	1 (2.6)
*SGA, n (%)	2,918 (20.0)	101 (23.9)	17 (44.7)
Apgar 1 min, median [IQR]	9 [8–9]	5 [4–7]	2 [1–3]
Apgar 5 min, median [IQR]	10 [10–10]	8 [7–9]	6 [2–7]
Early neonatal death, n (%)	63 (0.4)	20 (4.7)	17 (44.7)

*APH: antepartum haemorrhage; BMI: body max index; BW: birth weight; EGA: estimated gestational age at birth; LGA: large-for-gestational age; PROM: prolonged rupture of membranes; PTB: preterm birth; SGA: small-for-gestational age.

Maternal baseline characteristics included young mother (<20 years); underweight (BMI < 18.5 kg/m^2^); primigravida (first pregnancy); migration status (refugee and migrant women); literacy (self-reporting ability to read); smoking (yes/no); anaemia during pregnancy (haematocrit <30% in the last ANC visit); hypertension; malaria infection any time during pregnancy; presence of gestational diabetes (screening performed only from 2013 in high risk women at 24–26 gestational weeks), and previous neonatal death (yes/no).

Delivery baseline characteristics included prolonged rupture of membranes (>18h prior to delivery); prolonged second stage of labour (>1h); maternal fever (>38°C during labour or < 7 day’ prior to birth). Additional variables included the presence of antepartum haemorrhage (APH), breech delivery, fetal distress and meconium stained liquor.

Newborn baseline characteristics included sex; estimated gestational age (EGA) at birth (ultrasound in first ANC visit or use of the Dubowitz gestational age assessment for late ANC attenders.[12]); preterm birth (EGA at birth <37 weeks); small and large for gestational age (birthweight-for-gestational age < 10^th^ centile and > 90^th^ respectively in the Intergrowth-21^st^ Project size standard charts);[14] and low birth weight (<2500g at birth independently of EGA). Apgar score at 1 and 5 minutes after birth was recorded. Low Apgar at 5 min (score <6).

### Outcome measures

Early neonatal mortality (ENM) defined as newborn death occurring in the first 7 days of life was chosen as the measure of mortality. Neuro-developmental outcome at 1 year was available for a subset of children enrolled in several birth cohorts during the timeframe of this study. Neurodevelopment was evaluated with the standardised and validated Shoklo Developmental Test. The test evaluated 4 different aspects of development with each item scored as passed/failed (eye-hand coordination (maximum score 34), locomotor development (maximum score 24), speech (maximum score 6), and social interaction (maximum score 7)) [15]. A total score lower than 52 at one year of age identifies infants in need of attention or further investigation in this setting.[15] In addition, behavior during the test (relation to the tester, interest towards the test, emotional status) was evaluated (maximum score 15). Neuro-developmental follow-up was started in 2001 for children participating in other cohort studies, but is not routinely carried out on all newborns delivered at SMRU (supportive information, Text D in S1 File).

### Data management and quality

Data were routinely collected from the existing antenatal care (ANC), birth and special care baby unit (SCBU) paper records and stored electronically using Microsoft Access (Microsoft Corp, Redmond, WA, USA). Special effort was made to ensure that data entry was correct for newborns who had received resuscitation at birth by cross checking against paper records. Previous authors have highlighted the good quality and accuracy of the data collected at SMRU[16]. More than 95% of the cases had been entered correctly, and the remaining information was cleaned on a case-by-case basis. In total, 460 cases identified as having received neonatal resuscitation were included. These were reviewed by a paediatrician (SJ), and further classified into basic or advanced resuscitation.

### Statistical analysis

Statistical analysis was performed using STATA 14 for Windows (StataCorp, College Station, TX, USA). Kaplan-Meier survival curves were created to illustrate early neonatal mortality according to the need for resuscitation. The proportional hazards assumption was assessed visually and was not violated. Cox regression modelling was performed to estimate the association between early neonatal mortality and resuscitation, with adjustment for the potential confounders, age (<20 years), maternal anaemia, breech delivery, maternal fever, fetal distress, prematurity and low Apgar score at 5 min of life. Multivariable logistic regression modelling was performed to identify maternal and delivery factors associated with the odds of newborn resuscitation. To assess the association between newborn resuscitation and neurological developmental score at 10 to 15 months, multivariable linear regression analysis was performed with adjustment for prematurity, male newborn, maternal anaemia, maternal smoking, maternal literacy, maternal migrant status and breech delivery. Further details can be found in the supportive information, Text E in S1 File.

### Ethics statements

This study was approved by the Oxford Tropical Research Ethics Committee and the Faculty of Tropical Medicine at Mahidol University in Thailand granted ethical approval (OXTREC 585–16 and TMEC 16–020, respectively) and the Tak Province Community Ethics Advisory Board provided local permission (T-CAB-01/01/2016). All study data were extracted from Microsoft Access (Microsoft Corp, Redmond, WA, USA) database using a SMRU standard operating procedure. Data were fully anonymsed before analyses.

## Results

**Fig 2:**
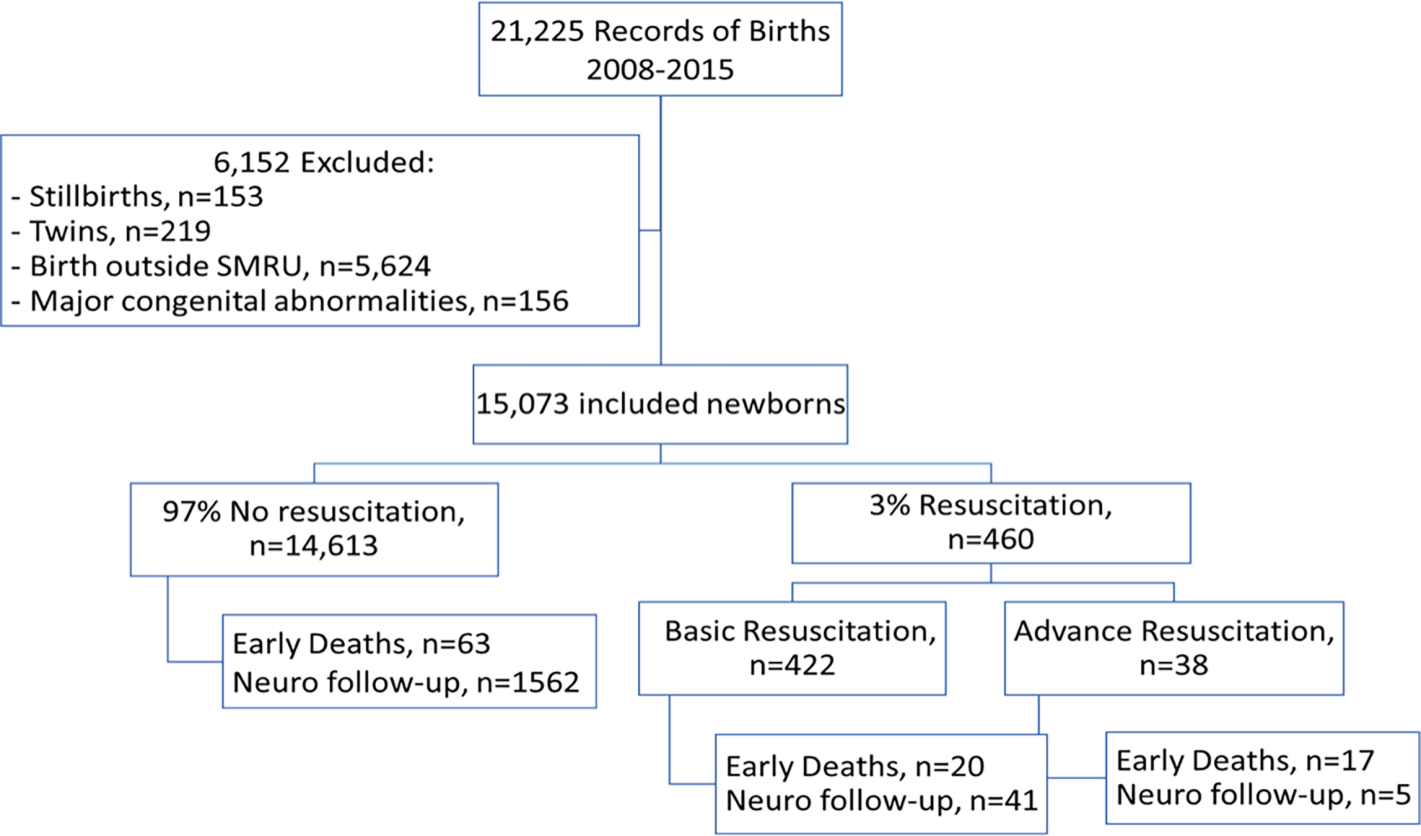
Flowchart of the study.

Between January 2008 and December 2015 there were 21,225 births recorded in the routinely collected antenatal care database. Of these, 6,152 records were excluded because they failed to meet the inclusion criteria. Records for 15,073 (71%) newborns were included in the analyses (Fig 2), of which 460 (3%) newborns received neonatal resuscitation at birth and, from those, 422 (2.7%, 95% CI (2.0–3.0)) underwent basic resuscitation and 38 (0.3%, 95% CI (0.1–0.3)) advanced resuscitation.

### Baseline characteristics of resuscitated and non-resuscitated newborns

Prolonged rupture of membranes, maternal fever, prematurity, small for gestational age and low birth weight <2500g, Apgar score at 1 and 5 min were associated with the level of resuscitation required (Table 1).

### Association between neonatal resuscitation and early neonatal mortality (ENM)

**Fig 3:**
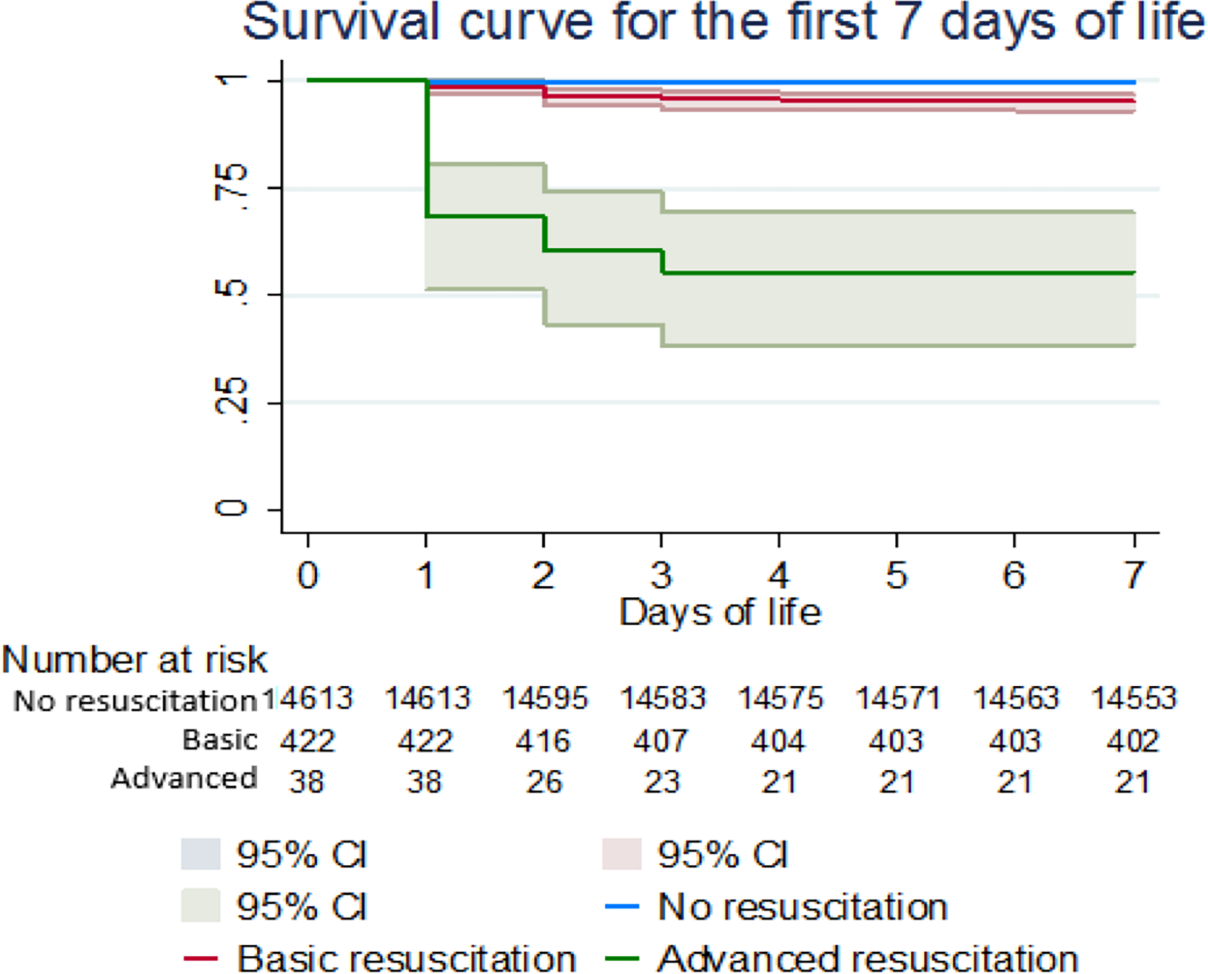
Kaplan Meier survival curve for early mortality according to the need for resuscitation.

Newborns receiving basic resuscitation presented a crude ENM rate of 47.4 per 1000 livebirths 95% CI (29.18–72.24), compared with 4.3 per 1000 livebirths 95% CI (3.31–5.51) for those in the no resuscitation group. A Kaplan-Meier curve is presented for no, basic and advanced resuscitation groups in Fig 3. All deaths (n = 17) in the advanced resuscitated group occurred in the first 72 hours after birth.

After adjusting for potential confounders, mortality in the basic resuscitation group was moderately increased compared with the no resuscitation group, although the confidence interval was wide (adjusted Hazard Ratio (HR) 1.30, 95% Confidence Interval (CI) (0.66–2.55), p value 0.442). Newborns requiring advanced resuscitation, however, remained more likely to die than non-resuscitated (adjusted HR 6.32, 95% CI (3.01–13.26), p<0.001) (Table 2).

**Table 2:** Association of early newborn death and any, basic and advanced resuscitation at birth.

	Deaths per number of newborns	Early Neonatal Mortality Rate per 1,000 livebirths (95% CI)	Univariable Analysis Hazard Ratio (95% CI), p-value	*Multivariable Analysis Hazard Ratio (95% CI), p-value
No resuscitation	63/14,613	4.3 (3.31–5.51)	1.00 (reference group)	1.00 (reference group)
Any resuscitation	37/460	80.4 (57.26–109.16)	19.39 (12.92–29.10), p<0.001	5.33 (2.83–10.03), p<0.001
Basic Resuscitation	20/422	47.4 (29.18–72.24)	8.84 (5.42–14.43), p<0.001	1.30 (0.66–2.55), p 0.442
Advanced Resuscitation	17/38	447.3 (286.24–617.00)	108.76 (64.39–183.69), p<0.001	6.32 (3.01–13.26), P<0.001

*Adjusted for confounders: maternal age (age<20 versus 20+ years), maternal anaemia, breech delivery, maternal fever, fetal distress, prematurity and low Apgar score at 5 min of life. Even though low birth weight (defined as <2500g) is a potential confounder this variable was not included in the multivariable model because prematurity is closely linked to birth weight, which is positively correlated with gestational age. Prematurity was chosen as the strongest factor influencing newborn death, in accordance to the published literature. [17,18]

### Neurodevelopmental score at one year of age

Among the 15,073 newborns in the cohort, 1,608 (10.5%) had a neurodevelopmental test at a median age of 12 months (interquartile range 12–12 months, range 11–14 months). Of these newborns, 1,562 had not been resuscitated, 41 had received basic, and 5 advanced resuscitation (Fig 2).

The median (IQR) neurodevelopmental test scores were comparable for non-resuscitated newborns and those that received basic resuscitation (Table 3). The five newborns who received advanced resuscitation had significantly lower test scores compared to non-resuscitated newborns (median score 56 (advanced resuscitation) versus 64 (no resuscitation), p = 0.017).

**Table 3:** Median neurological developmental test scores for followed cohort at 10 to 15 months of age.

Neurological outcome score	No Resuscitation N = 1565	Basic N = 41	Advanced N = 5**
Total Shoklo score, median [IQR] (range)	64 [61–65] (15–70)	63 [59–65] (26–69)	56 (21–64)
Coordination score, median [IQR] (range)	31 [30–32] (3–34)	30.5 [30–32] (15–34)	30 (9–31)
Motor score, median [IQR] (range)	21 [20–23] (5–24)	20.5 [19–21] (4–24)	17 (6–23)
Speech score, median [IQR] (range)	5 [4–5] (1–6)	5 [4–5] (2–6)	5 (2–5)
Social score, median [IQR] (range)	7 [6–7] (1–7)	7 [6–7] (4–7)	6 (4–7)
Behaviour score, median [IQR] (range)	15 [15–15] (3–15)	15 [14–15] (12–15)	15 (12–15)

IQR–inter-quartile range

** IQR was omitted as the advanced resuscitation group has small numbers

### Perinatal risk factors associated with neonatal resuscitation

**Fig 4:**
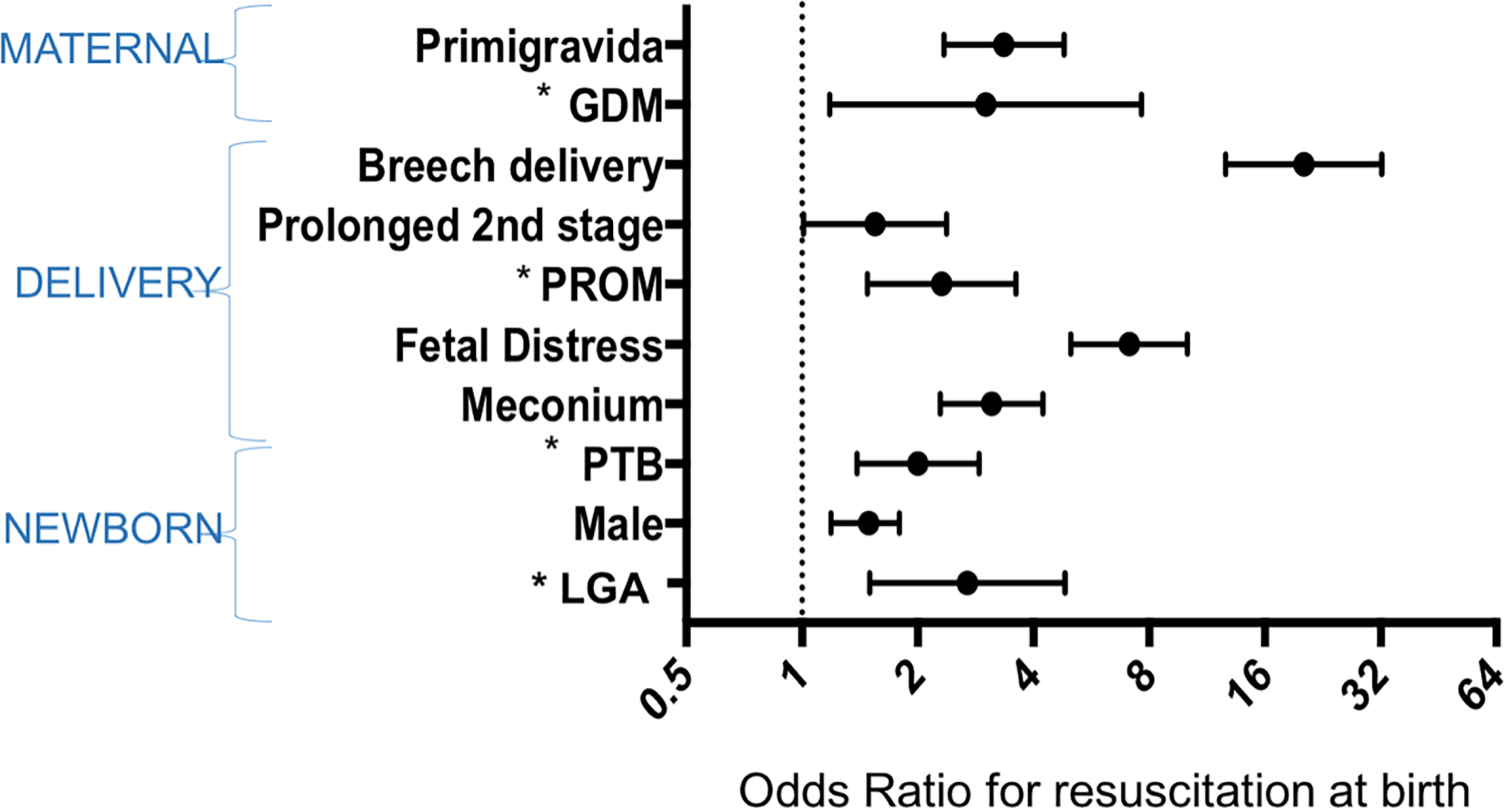
Maternal, delivery and newborn risk factors associated with neonatal resuscitation at birth. **Adjusted Odds Ratio and 95% CI in log scale. *GDM, gestational diabetes mellitus; PROM, prolonged rupture of membranes; PTB, preterm birth; LGA: large-for-gestational age. ** Odds of requiring resuscitation at birth estimated from three separate multivariable logistic regression models: maternal factors; delivery factors–adjusted for maternal confounders; newborn factors adjusted for maternal and delivery confounders.

Vaginal breech newborns were 20.1 times (95% CI 12.65-32-22, p-value <0.001) more likely to need resuscitation at birth compared with normal cephalic vaginal deliveries (Fig 4). Other predictors of neonatal resuscitation were primigravida, prolonged rupture of membranes (>18hrs), prolonged second stage of labour, fetal distress, meconium, preterm birth, large for gestational age and newborn male sex (Fig 4; Unadjusted and adjusted ORs (95% CI) values given in supportive information S1 Table).

## Discussion

This retrospective study has explored the outcomes of neonatal resuscitation at SMRU. Early neonatal mortality was examined in three groups: newborns who were not resuscitated at birth, those who received basic resuscitation, and those who received advanced resuscitation. The difference in mortality observed between low-risk newborns and those who received resuscitation at birth was mostly related to their adverse baseline characteristics, such as maternal age (young mothers aged<20), maternal anaemia, breech delivery, maternal fever, fetal distress, prematurity and low Apgar score at 5 min of life. In the smaller cohort of infants followed at one year of age those who had received basic resuscitation at birth had a normal neuro-developmental score compared to those who were not resuscitated. The identification of risk factors for resuscitation and poor neonatal outcomes, such as breech delivery or prematurity, facilitates the provision of targeted preventive measures.

### Early neonatal mortality

Compared to worldwide figures, the SMRU early neonatal mortality rate is similar to reports from middle-income countries such as Thailand (neonatal mortality rate of 6.7 per 1000 livebirths in 2015 [19]). High-risk deliveries are referred onto the Thailand public hospital system and are not, therefore, included in this analysis. There still remains a significant gap given the lower neonatal mortality observed in high-income countries, such as the United Kingdom with an ENM rate of 2.4 per 1000 livebirths.[19]

The crude ENM rate by the different groups of interest in the study showed an ENM rate of 47 per 1000 live births in the basic resuscitation group and a 100-fold increase (447 per 1000 live births) in the newborns requiring advanced resuscitation. ENM rate depends on multiple factors, and the crude ENM rate in both groups reflects their different baseline characteristics including younger maternal age (age<20 years), maternal anaemia, breech delivery, maternal fever, fetal distress, prematurity and low Apgar score at 5 min of life. When adjusting for such differences, the association between basic resuscitation and early neonatal death failed to reach statistical significance (HR: 1.30; 95% CI (0.66–2.55), p = 0.442) and the increased risk of death in the advanced resuscitation group fell from 108.7 to 6.3; 95% CI (3.01–13.26), p<0.001. This attenuation highlights the importance of addressing other factors that play an important role in neonatal mortality and endorses continuum of maternal and newborn care as a relevant strategy to tackle newborn health.

### Neurodevelopmental outcomes

It is well recognised that moderate to severe birth hypoxia can have long-term consequences.[10] Evaluating these consequences in low-resource settings remains a major challenge, and the burden of children with disabilities has not been addressed in the majority of resource-limited countries. In this study, the findings are important, since they show that children who had received basic resuscitation immediately after birth had comparable neuro-development scores to non-resuscitated children. Despite the limited number of children who had neuro-developmental testing at the end of the first year of life, the findings suggest, reassuringly, that the provision of basic resuscitation at SMRU has not increased the burden of disability at one year. This is motivating for front-line staff, and supports the case for rolling out and strengthening basic neonatal resuscitation at all health facilities where births occur. Although it appears that newborns given advanced resuscitation have neuro-developmental scores in the low-normal range at one year, this sub-group was very small, limiting meaningful interpretation. Few studies have described the neurological outcomes of infants who required resuscitation at birth in low-resource settings, however the scarce evidence available reported similar findings[20–22] and encourages the use of basic resuscitation in such settings. It is important to emphasise that neuro-developmental delays such as cerebral palsy might not be visible in the first year of life, as observed by Squarza et al., who found in extremely low birth weight children an increased number of learning disabilities at school age.[23] For this reason, the findings of this research need to be interpreted with caution, as normal neurological scores at one year of age do not imply the absence of neuro-developmental delays in the longer term. Data from middle and low income settings on the identification of minor cognitive deficits, behavioural problems and longer follow-up periods (school age) in resuscitated children at birth are still lacking. Future prospective studies with longer follow-up period might help to clarify this scenario.

The use of adrenaline in low resource setting is debatable when the possibility for intubated ventilation or additional intensive care is not available. In this study, 45% (17/38) of the newborns requiring advanced resuscitation died. From the 21 survivors only 5 were followed up to one year. These small numbers prohibit meaningful interpretation of the neurodevelopmental scores in this group of infants. Future studies could examine the long-term consequences of advanced resuscitation in low-resource settings, and assess whether the use of adrenaline is appropriate.

### Risk factors predicting an increased need for neonatal resuscitation

Identification of baseline characteristics that increase the need for neonatal resuscitation is useful for frontline staff planning the deployment of human resources for high-risk deliveries. In places where skilled human resources are scarce, effective allocation of skilled staff is the cornerstone of providing good quality-care. The risk factors identified in this study (Fig 4) are concordant with those described in the literature.[24] Nonetheless, increased allocation of staff is not always possible, and high staff turnover makes it difficult to keep all staff attending deliveries updated in neonatal resuscitation. The implementation of a triage system that classifies pregnancies and deliveries into high, moderate and low risk of requiring neonatal resuscitation, could improve the allocation of skilled health workers. Although this prioritising system is currently in place at SMRU, this study reinforces current good practice and adds evidence to the field.

### Limitations

The main limitation of this study is the retrospective design, which limits the capacity to identify the added value that neonatal resuscitation has in reducing early neonatal mortality. The small number of newborns who participated in neuro-developmental follow-up reduces the power of the analysis to detect subtle differences between groups, and, in the case of advanced resuscitation, prohibits meaningful statistical analysis of this sub-group. Nonetheless, the comprehensive data collection system, high quality data, small loss to follow-up, accurate gestational age assessment and standardised neuro-developmental follow-up, mean that this dataset provides us with some valuable insights.

### Future research

There are still gaps in the evidence regarding the impact and outcomes of neonatal resuscitation in low-resource settings. Future studies evaluating the appropriate level of resuscitation will help to ensure adequate allocation of staff and to identify the equipment and skills needed in those settings. Other areas to explore are the assessment of minor cognitive delay and behaviour in children who received resuscitation at birth and the long-term neurological outcomes of children who received advanced resuscitation. These studies will help to identify the proportion of high-risk children that will require long-term support in such settings.

## Conclusions

Basic resuscitation by locally trained health workers results in comparable neuro-development outcomes at one year of age to newborns who did not require resuscitation. This finding supports efforts to scale-up neonatal resuscitation and provide continuous training to maintain bag-and-mask ventilation skills in resource-limited settings, without the risk of increasing the burden of long-term disabilities. The identification of baseline characteristics that predict an increased risk of requiring neonatal resuscitation could be used by frontline staff to guide allocation of human resources for high-risk deliveries. It is important to acknowledge that neonatal resuscitation is only one intervention in the continuum of care, and, in order to reduce neonatal mortality, a multipronged strategy needs to be adopted in the places where most neonatal deaths occur.

## Supporting information

S1 File **Text A.** The setting: Shoklo Malaria Research Unit (SMRU). **Text B.** Criteria for performing neonatal resuscitation. **Text C.** Selection of the variables included in the study. **Text D.** The Shoklo Developmental test. **Text E.** Additional statistical analysis.
(DOCX)

S1 Fig Causal diagram. (TIFF)

S1 Table Association between maternal, delivery and newborn characteristics with requiring neonatal resuscitation at birth. *APH: antepartum haemorrhage; LBW: Low birth weight; LGA: large-for-gestational age; PROM: prolonged rupture of membranes; PTB: preterm birth; SGA: small-for-gestational age.
** Low birth weight (<2,500g) and SGA variables were not included in the final multivariable model because these are collinear with prematurity.
***Separate models were fitted for maternal, delivery and newborn characteristics with adjustment for maternal confounders, maternal and delivery confounders, and maternal, delivery and newborn confounders in each model respectively.
(DOCX)
