# Supplementary material for: Early neonatal mortality and neurological outcomes of neonatal resuscitation in a resource-limited setting on the Thailand-Myanmar border: A descriptive study
Source: PLoS One. 2018 Jan 5;13(1):e0190419. doi: 10.1371/journal.pone.0190419 (PMC5755780; doi:10.1371/journal.pone.0190419)
Supplement: S1 File — Text A. The setting: Shoklo Malaria Research Unit (SMRU). Text B. Criteria for performing neonatal resuscitation. Text C. Selection of the variables included in the study. Text D. The Shoklo Developmental test. Text E. Additional statistical analysis. (DOCX) [file pone.0190419.s001.docx]

**Supportive information**

**S1 File. Text A. The setting: Shoklo Malaria Research Unit (SMRU)**

The Shoklo Malaria Research (SMRU) was established in 1986, located at the Shoklo refugee camp on the Thailand-Myanmar. At the time of the analysis of this data SMRU was comprised of three rural clinics: Mae La refugee camp, and two migrant sites, Mawker Tai and Wang Pha. The three sites provide maternity services to the refugee and migrant Burmese women. Each site has a delivery room staffed by locally trained birth attendants; approximately 2,500 babies are delivered each year in total across the SMRU clinics. Pregnant women are seen regularly throughout pregnancy. Over the years, SMRU has successfully implemented targeted interventions to reduce maternal, neonatal and child mortality [1–3].

An effort to improve the quality neonatal care was pursued in 2008 with the opening of a dedicated area to care for unwell neonates, named the special care baby unit (SCBU). Locally appropriate standardised neonatal guidelines were developed and training of the staff was done locally with good results as shown by White et al [4]. Training was focused on recognition of the sick neonate, resuscitation of the neonate, examination of a neonate including routine newborn examination, supportive medical care, specific medical care and of counselling parents with premature or sick newborns [1].

Since the opening of SCBU, neonatal resuscitation training has been established and standardised at all 3 sites, with continuous training on a yearly basis with Advanced Life Support in Obstetrics®(ALSO®) certification. Despite, continuous training and adequate implementation on neonatal resuscitation some limitations still exist as there is no capacity for intubation, mechanical ventilation or insertion of umbilical lines. Bag-and-mask ventilation, chest compression and adrenaline are used during resuscitation, but resuscitation outcomes have not been analysed.

**S1 File. Text B. Criteria for performing neonatal resuscitation**

Neonatal resuscitation was performed by local staff at all three SMRU sites. The decision to commence neonatal resuscitation at birth was based on the clinical judgement of the frontline staff, who follow the SMRU standardised guideline, aligned to ALSO® guidelines. Basic resuscitation was initiated in those newborns who had not established regular breathing within one minute of birth. Apgar scores were recorded at 1 and 5 minutes. Advanced resuscitation was commenced if the heart rate remained persistently below 60 bpm despite adequate ventilation.

**S1 File. Text C. Selection of the variables included in the study**

The maternal, delivery and neonatal baseline variables were chosen after a causal diagram was created (S1 Fig), based on an overview of the published literature [5–7]. The causal diagram shows the interrelationship between maternal, delivery and newborn characteristics that play a role in the need for resuscitation, influencing at the same time the neuro-developmental score and early neonatal death.

**S1 File. Text D. The Shoklo Developmental test**

The Shoklo Developmental Test was validated in infants in the refugee camp and in London [8]. It was specifically developed for resource constrained settings and where limited English skills form a barrier to neurodevelopmental testing in young children. Inter-tester quality control tests showed a high level of agreement of 95% and testers who are trained on site, undergo regular quality control activities.

**S1 File. Text E. Additional statistical analysis**

Baseline characteristics of resuscitated and non-resuscitated newborns were described by using mean and standard deviation for maternal age, estimated gestational age in weeks and birth weight variables; and median and inter-quartile for Apgar score; and n(%) for categorical variables. Maternal, delivery and newborn variables were compared in each study group using the Chi-squared test or the Fisher’s exact test, as appropriate, with the Bonferroni correction being applied for multiple comparisons. The Chi-square test for trend was used to compare baseline characteristics of the 3 study groups (no resuscitation, basic resuscitation and advanced resuscitation). Continuous variables were compared using t-test. The Mann-Whitney U test was used to compare neurological outcome scores between no resuscitation and basic and advanced resuscitation groups.

Logistic regression was used to analyse the association between maternal, delivery and newborn characteristics with requiring neonatal resuscitation at birth. Separate models were fitted for maternal, delivery and newborn characteristics with adjustment for maternal confounders, maternal and delivery confounders, and maternal, delivery and newborn confounders in each model respectively.

**References**

1. Turner C, Carrara V, Aye N, Thein M, Chit N, Mo M, et al. Neonatal Intensive Care in a Karen Refugee Camp : A 4 Year Descriptive Study. PLoS One. 2013;8: 1–9. doi:10.1371/journal.pone.0072721

2. Mcgready R, Boel M, Rijken MJ, Ashley EA, Cho T, Moo O, et al. Effect of Early Detection and Treatment on Malaria Related Maternal Mortality on the North-Western Border of Thailand 1986 – 2010. PLoS One. 2012;7. doi:10.1371/journal.pone.0040244

3. Hoogenboom G, Thwin MM, Velink K, Baaijens M, Charrunwatthana P, Nosten F, et al. Quality of intrapartum care by skilled birth attendants in a refugee clinic on the Thai-Myanmar border : a survey using WHO Safe Motherhood Needs Assessment. BMC Pregnancy Childbirth. 2015;15: 1–9. doi:10.1186/s12884-015-0444-0

4. White AL, Min TH, Gross MM, Kajeechiwa L, Thwin MM, Hanboonkunupakarn B, et al. Accelerated training of skilled birth attendants in a marginalized population on the Thai-myanmar border: A multiple methods program evaluation. PLoS One. 2016;11: 1–18. doi:10.1371/journal.pone.0164363

5. Aziz K, Chadwick M, Baker M, Andrews W. Ante- and intra-partum factors that predict increased need for neonatal resuscitation. Resuscitation. 2008;79: 444–452. doi:10.1016/j.resuscitation.2008.08.004

6. Ozturk A, Demirci F, Yavuz T, Avsar Y, Dosoglu M. Antenatal and delivery risk factors and prevalence of cerebral palsy in Duzce ( Turkey ). Brain Dev. 2007;29: 39–42. doi:10.1016/j.braindev.2006.05.011

7. Lawn JE, Cousens S, Zupan J. 4 Million neonatal deaths: When? Where? Why? Lancet. 2005;365: 891–900. doi:10.1016/S0140-6736(05)71048-5

8. Haataja L, Mcgready R, Arunjerdja R, Simpson JA, Mercuri E, Dubowitz L. A new approach for neurological evaluation of infants in resource-poor settings. Ann Trop Paediatr. 2002;22: 355–368. doi:10.1179/027249302125002029
