# Supplementary figures and images for: Early neonatal mortality and neurological outcomes of neonatal resuscitation in a resource-limited setting on the Thailand-Myanmar border: A descriptive study
Source: PLoS One. 2018 Jan 5;13(1):e0190419. doi: 10.1371/journal.pone.0190419 (PMC5755780; doi:10.1371/journal.pone.0190419)

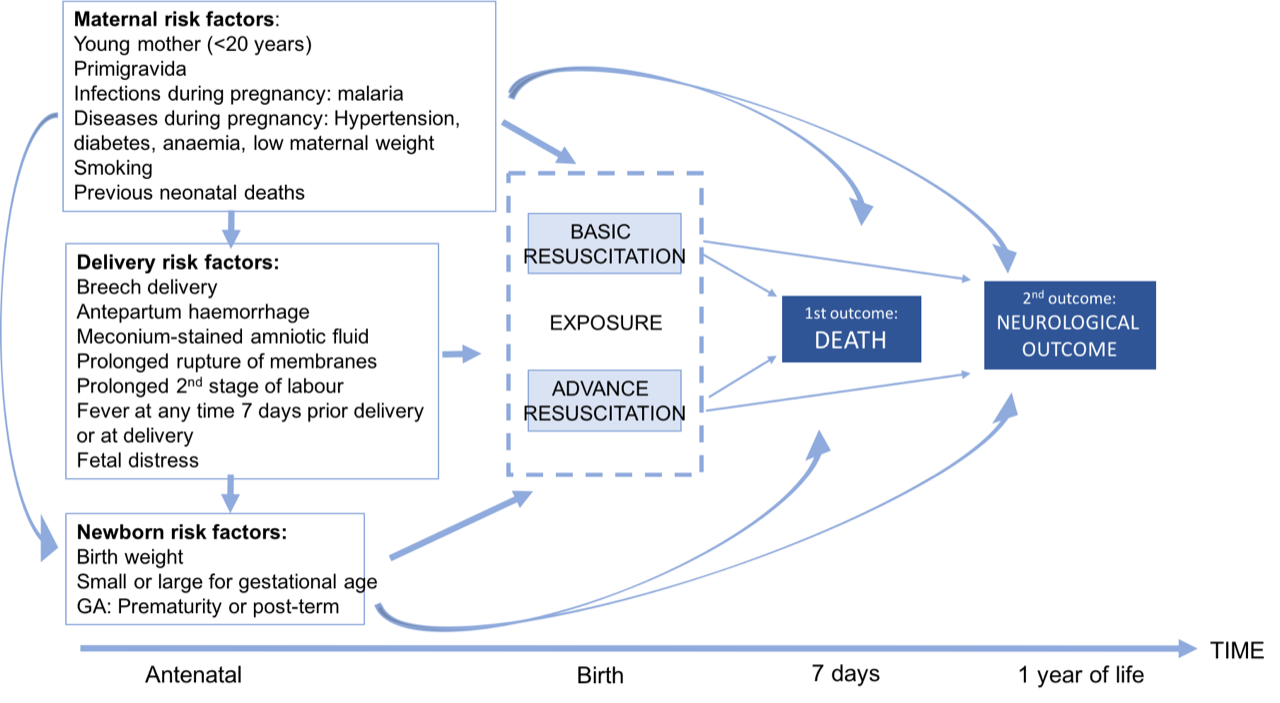

Supplement: S1 Fig — (TIFF) [file pone.0190419.s002.tiff]
