# Supplementary material for: Early neonatal mortality and neurological outcomes of neonatal resuscitation in a resource-limited setting on the Thailand-Myanmar border: A descriptive study
Source: PLoS One. 2018 Jan 5;13(1):e0190419. doi: 10.1371/journal.pone.0190419 (PMC5755780; doi:10.1371/journal.pone.0190419)
Supplement: S1 Table — *APH: antepartum haemorrhage; LBW: Low birth weight; LGA: large-for-gestational age; PROM: prolonged rupture of membranes; PTB: preterm birth; SGA: small-for-gestational age. ** Low birth weight (<2,500g) and SGA variables were not included in the final multivariable model because these are collinear with prematurity. ***Separate models were fitted for maternal, delivery and newborn characteristics with adjustment for maternal confounders, maternal and delivery confounders, and maternal, delivery and newborn confounders in each model respectively. (DOCX) [file pone.0190419.s003.docx]

**S 1 Table.** **Association between maternal, delivery and newborn characteristics with requiring neonatal resuscitation at birth**

|  | **Livebirths**  **N=** **15073** | **Odds Ratio for neonatal resuscitation**  **Univariable Analysis**  **OR (95% CI), p-value** | **Odds Ratio for neonatal resuscitation**  *****Multivariable Analysis**  **OR (95% CI), p-value** |
| --- | --- | --- | --- |
| **Maternal**  **Characteristics** |  |  |  |
| Young mother, n (%) | 2,705 (17.95) | 1.78 (1.44- 2.19)  p <0.001 | 1.16 (0.82-1.64)  p= 0.385 |
| Maternal low weight  (BMI < 18.5 kg/m^2^) | 1,511 (10.27) | 1.05 (0.77-1.42)  p =0.739 | 1.17 (0.74-1.85)  p =0.486 |
| Primigravida n, (%) | 5,101 (33.84) | 1.23 (0.86- 1.77)  p =0.251 | 3.35 (2.34- 4.80)  p<0.001 |
| Migrant n, (%) | 7,013/ 15,072 (46.53) | 0.85 (0.73- 1.12)  p =0.368 | 0.84 (0.62- 1.13)  p =0.257 |
| Literate n, (%) | 7,635/ 12,168 (62.75) | 1.05 (0.84- 1.31)  p =0.631 | 0.72 (0.53- 0.99)  p =0.046 |
| Smoking n, (%) | 2,134 (14.16) | 0.71 (0.53- 0.96)  p =0.029 | 0.92 (0.54-1.58)  p =0.776 |
| Anaemia n, (%) | 625/ 15,039 (4.16) | 0.82 (0.49- 1.37)  p =0.460 | 0.99 (0.39- 2.45)  p =0.98 |
| Any maternal hypertension n, (%) | 674 (4.47) | 1.85 (1.31- 2.63)  p<0.001 | 1.02 (0.47-2.22)  p =0.94 |
| Malaria n, (%) | 399 (2.65) | 0.91 (0.63- 1.31)  p =0.631 | 0.34 (0.04- 2.53)  p =0.297 |
| Gestational diabetes n, (%) | 139/ 7,705 (1.8) | 1.39 (0.56- 3.44)  p =0.470 | 3.01 (1.18-7.63)  p =0.020 |
| Previous neonatal death n, (%) | 4,346/ 14,982 (29.0) | 0.90 (0.73- 1.12)  p =0.368 | 1.03 (0.69- 1.53)  p =0.868 |
| **Delivery Characteristics** |  |  |  |
| *APH n, (%) | 38/15,048 (0.25) | 1.79 (0.43- 7.48)  p =0.421 | 1.63 (0.13-19.97)  p =0.699 |
| Breech delivery n, (%) | 228 (1.51) | 17.59 (13.08-23.65)  p<0.001 | 20.19 (12.65-32.22)  p <0.001 |
| Prolonged 2^nd^ stage n, (%) | 972 (6.45) | 3.56 (2.79 - 4.54)  p<0.001 | 1.55 (1.01-2.38)  p =0.043 |
| *PROM n, (%) | 642/ 14,659 (4.38) | 2.12 (1.50- 2.99)  p<0.001 | 2.31 (1.48-3.60)  p <0.001 |
| Fever n, (%) | 237 (1.58) | 3.59 (2.31- 5.58)  P<0.001 | 1.53 (0.71- 3.28)  p =0.270 |
| Foetal distress n, (%) | 702 (4.66) | 2.42 (1.35- 4.32)  p =0.005 | 7.10 (5.00-10.06)  p<0.001 |
| Meconium n, (%) | 3,723 (24.70) | 3.07 (2.54- 3.70)  P<0.001 | 3.11 (2.29-4.23)  p<0.001 |
| **Newborn Characteristics** |  |  |  |
| *PTB n, (%) | 720 (4.78) | 2.59 (1.92- 3.51)  p <0.001 | 2.00 (1.39-2.89)  p <0.001 |
| Male n, (%) | 7,792 (51.70) | 1.44 (1.19- 1.74)  p<0.001 | 1.46 (1.19-1.79)  p<0.001 |
| *LBW <2500g n, (%) | 1,877 (12.45) | 2.11 (1.69- 2.64)  p <0.001 | **Not included in the final multivariable model |
| *LGA n, (%) | 281 (1.86) | 1.55 (0.88- 2.73)  p =0.124 | 2.69 (1.50-4.83)  P<0.001 |
| *SGA n, (%) | 3,036 (20.14) | 1.38 (1.11- 1.71)  p =0.003 | **Not included in the final multivariable model |

*APH: antepartum haemorrhage; LBW: Low birth weight; LGA: large-for-gestational age; PROM: prolonged rupture of membranes; PTB: preterm birth; SGA: small-for-gestational age

** Low birth weight (<2,500g) and SGA variables were not included in the final multivariable model because these are collinear with prematurity.

***Separate models were fitted for maternal, delivery and newborn characteristics with adjustment for maternal confounders, maternal and delivery confounders, and maternal, delivery and newborn confounders in each model respectively.
